# Better implant survival with modern ankle prosthetic designs: 1,226 total ankle prostheses followed for up to 20 years in the Swedish Ankle Registry

**DOI:** 10.1080/17453674.2019.1709312

**Published:** 2020-01-13

**Authors:** Alexandra Undén, Lars Jehpsson, Ilka Kamrad, Åke Carlsson, Anders Henricson, Magnus K Karlsson, Björn E Rosengren

**Affiliations:** a Department of Radiology, Skåne University Hospital, Malmö;; b Department of Clinical Sciences Malmo (IKVM), Lund University;; c Department of Orthopedics, Skåne University Hospital, Malmö;; d Department of Orthopedics, Falu Central Hospital and Center of Clinical Research Dalarna, Falun, Sweden

## Abstract

Background and purpose — We have previously reported on the prosthetic survival of total ankle replacements (TAR) in Sweden performed between 1993 and 2010. Few other reports have been published on 5- and 10-year survival rates. Furthermore, there is a lack of long-term outcome data on modern prosthetic designs. Therefore, we compared early and current prosthetic designs after a mean 7-year follow-up.

Patients and methods — On December 31, 2016, 1,230 primary TARs had been reported to the Swedish Ankle Registry. We analyzed prosthetic survival, using exchange or permanent extraction of components as endpoint for 1,226 protheses with mean follow-up of 7 years (0–24). Differences between current (Hintegra, Mobility, CCI, Rebalance, and TM Ankle) and early prosthetic designs (STAR, BP, and AES) were examined by log rank test.

Results — 267/1,226 prostheses (22%) had been revised by December 31, 2016. We found an overall prosthetic survival rate at 5 years of 0.85 (95% CI 0.83–0.87), at 10 years 0.74 (CI 0.70–0.77), at 15 years 0.63 (CI 0.58–0.67), and at 20 years 0.58 (CI 0.52–0.65). For early prosthetic designs the 5- and 10-year survival rates were 0.81 (CI 0.78–0.84) and 0.69 (CI 0.64-0.73) respectively, while the corresponding rates for current designs were 0.88 (CI 0.85–0.91) and 0.84 (CI 0.79–0.88). Current prosthetic designs had better survival (log rank test p < 0.001).

Interpretation — Our results point to a positive time trend of prosthetic survival in Sweden; use of current prosthetic designs was associated with better prosthetic survival. Improved designs and instrumentation, more experienced surgeons, and improved patient selection may all have contributed to the better outcome.

Uncemented, 3-component total ankle replacements (TAR) have shown promising but somewhat varying results in medium- and long-term reports (Wood and Deakin 2003, Fevang et al. 2007, Skytta et al. 2010, Bonnin et al. 2011, Henricson et al. 2011b, Mann et al. 2011, Tomlinson and Harrison 2012, Barg et al. 2013, Zaidi et al. 2013, Henricson and Carlsson 2015, Kerkhoff et al. 2016, Frigg et al. 2017, Palanca et al. 2018, Clough et al. 2019). Some evidence points to better results with modern prosthetic designs (Barg et al. 2015, Koivu et al. 2017a, Clough et al. 2019). National registries give better insight into current real-world results, including more patients and different surgeons, than data from single surgeons or institutions. 5-year survival rates of between 0.78 and 0.89 have been reported by national registries from Finland, New Zealand, Norway, and Sweden (Fevang et al. 2007, Henricson et al. 2007, Hosman et al. 2007, Skytta et al. 2010, Henricson et al. 2011b). Results beyond 5 years are uncertain. Here we present medium and longer-term follow-up (up to 20 years) of TARs reported to the Swedish Ankle Registry (http://www.swedankle.se), and compare early and current designs.

## Patients and methods

Since 1993, Swedish hospitals performing TARs have reported information on date of index surgery and any revision surgery, including data on the patient and the procedure, to the Swedish Ankle Registry. The current procedure-based coverage and completeness are both estimated at close to 100%.

Until December 31, 2016, 1,230 primary TARs (all uncemented, 3-component designs) had been registered in 1,132 patients with mean annual numbers of 51 (6–87). The mean follow-up time was 7 years (0–24). 4 cases lost to follow-up were not included in the survival analyses. 546 of the remaining 1,226 cases we refer to as “early prosthetic designs” (STAR, BP, and AES) as these designs have not been implanted in Sweden since 2008; the other 680 we refer to as “current prosthetic designs” (Hintegra, Mobility, CCI, Rebalance, and TM Ankle) (Table 1). Revision was defined as removal or exchange of 1 or more of the prosthetic components with the exception of incidental exchange (exchange of the polyethylene insert during a secondary procedure undertaken because of a different indication) of the polyethylene insert (Henricson et al. 2011a). We chose to analyze the number of prostheses rather than the number of patients (including 96 bilateral cases), in line with our previous study (Henricson et al. 2011b) as this approach has been found to have a negligible effect on the survival estimates (Ranstam and Robertsson 2010).

**Table 1.** Distribution of TARs implanted in Sweden during 1993–2016 by year of implantation and prosthetic design

	Early prosthetic designs				Current prosthetic designs				
Year	STAR **^a^**	BP **^b^**	AES **^c^**	Hintegra **^d^**	Mobility **^e^**	CCI **^f^**	Rebalance **^g^**	TM Ankle **^h^**	Total
1993	6								6
1994	13								13
1995	11								11
1996	8								8
1997	24								24
1998	34								34
1999	25	1							25
2000	45								46
2001	48	21	3	10					48
2002	39	23	17	14					73
2003	25	29	16	4					79
2004	18	13	23	2	9				67
2005	18	11	21	6	21				65
2006	8	7	18		20				67
2007	1	4	17		32	16			46
2008					31	42			69
2009					41	22			73
2010					44	22	21		63
2011					23	31	27		87
2012					32	12	34		81
2013					15	4	37	5	78
2014					1	2	36	11	61
2015				3			23	21	53
2016				9					53
Total	323	109	115	48	269	151	178	37	1,230

a Scandinavian Total Ankle Replacement (Waldemar LINK GmBH, Hamburg, Germany)

b Buechel–Pappas (Wright Cremascoli, Toulon, France)

c Ankle Evolutive System (Biomet, Valence, France)

d Hintegra (Newdeal SA, Lyon, France)

e Mobility (DePuy International, Leeds, UK)

f CCI–Ceramic Coated Implant (Wright Medical Technology, Arlington)

g Rebalance (Biomet, Bridgend, UK)

h Trabecular Metal Total Ankle (Zimmer inc, Warsaw, Indiana, USA)

Since 1993, TAR has been performed in 25 hospitals in Sweden by 43 surgeons. In the total cohort, 60% were women and the mean age at primary TAR was 60 years (18–88). The most common primary diagnoses leading to surgery were posttraumatic arthritis in one-third of patients and rheumatoid arthritis in one-third (Table 2).

**Table 2.** Data on distribution of diagnoses, sex and age per prosthetic design group

Design group Diagnosis	n (%)	women (%)	Age mean (SD) [range]
All prosthetic designs			
Posttraumatic arthritis	443 (36)	53	60 (12) [25–86]
Rheumatoid arthritis	401 (32)	81	56 (14) [18–85]
Osteoarthritis	291 (24)	46	64 (11) [30–88]
Other^a^	95 (8)	40	59 (12) [28–76]
All diagnoses	1,230	60	60 (13) [18–88]
Current prosthetic designs			
Posttraumatic arthritis	268 (39)	52	62 (12) [30–85]
Rheumatoid arthritis	182 (27)	87	57 (14) [18–83]
Osteoarthritis	155 (23)	45	67 (10) [37–88]
Other^a^	78 (11)	37	60 (11) [34–76]
All diagnoses	683	58	62 (12) [18–88]
Early prosthetic designs			
Posttraumatic arthritis	175 (32)	54	56 (12) [25–86]
Rheumatoid arthritis	219 (40)	77	56 (14) [21–85]
Osteoarthritis	136 (25)	48	61 (10) [30–84]
Other^a^	17 (3)	53	53 (15) [28–74]
All diagnoses	547	62	57 (13) [21–86]

a Including hemophilia, hemochromatosis and psoriatic arthritis

### Statistics

To visualize differences in prosthetic survival rate, we used Kaplan–Meier estimator. Differences between current and early prosthetic designs were examined by log rank test.

Data are reported as numbers and proportions (%), mean (SD), or median (range). We considered a probability of less than 5% as statistically significant and used 95% confidence intervals (CI) within parentheses to describe uncertainty.

Even though our study may not be considered sample based we have chosen to present measure of uncertainty in order to facilitate generalization to probable future outcomes in Sweden and to other similar populations. We used SPSS version 23 (IBM Corp, Armonk, NY, USA).

### Ethics, data sharing, funding, and potential conflicts of interest

The study protocol was approved by the Ethical

Review Board of Lund University (Dnr 2014/448). The study was conducted in accordance with the Helsinki

Protocol.

The registration of data and the study was performed confidentially on patient consent and according to Swedish and EU data protection rules. Data may be accessible upon application to the registry.

This work was supported by grants from ALF and FoUU of Region Skåne, Greta Koch, Herman Järnhardt, Maggie Stevens, Skåne University Hospital foundations, and the Swedish Association of Local Authorities and Regions. The funders had no influence on the design of the study, the collection, analysis, and interpretation of data, on writing the manuscript, or on any other part of the study.

The authors declare no conflict of interest.

## Results

Of the 1,230 prostheses implanted since 1993, 22% had been revised by December 31, 2016. The most common reason for revision was loosening of the tibial and/or the talar component, responsible for about half of the revisions in both the early design group and current design group. PE (polyethylene) insert failure (13%) was the second most common reason for revision in the early design group, whereas this was only reported once in the current design group (Table 3).

**Table 3a.** Information on reasons for revision for early prosthetic designs

Reasons for revision	STAR n = 323	BP n = 109	AES n = 115	Total (%)n = 547
Talus and/or tibia				
loosening	65	7	16	88 (45)
Technical error	17 **^a^**	2	19 (10)	
Instability	1	3	3	7 (4)
Infection	16	1	4	21 (11)
Intractable pain	10	1	1	12 (6)
PE failure	22	2	2	26 (13)
Painful varus	2	2	2	6 (3)
Painful valgus		1	4	5 (3)
Fracture/dislocation	3	3	2	8 (4)
Other			5	5 (3)
Total number of revisions (%)	136 (42)	22 (20)	39 (34)	197 (36)

**Figure 1.**
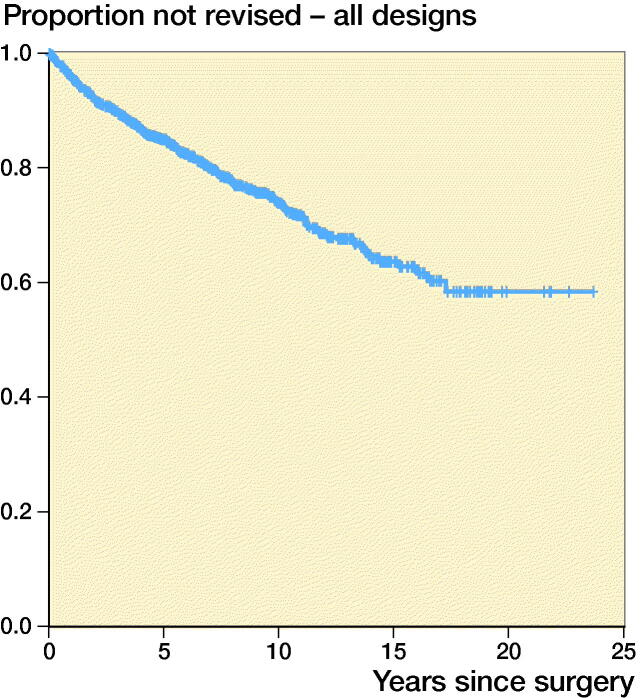
Estimated cumulative prosthetic survival for all 1,226 TARs. Number of patients still at risk of experiencing the primary endpoint and prosthetic survival with 95% CI per 5-year period are indicated in the life table

We found an overall TAR survival at 5 years of 0.85 (95% CI 0.83–0.87), at 10 years 0.74 (CI 0.70–0.77), at 15 years 0.63 (CI 0.58–0.67) and at 20 years 0.58 (CI 0.52–0.65) (Figure 1).

**Figure 2.**
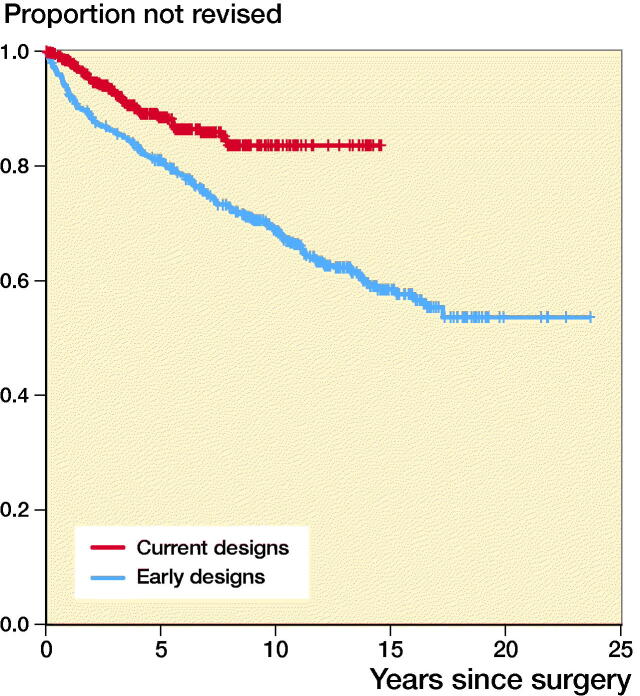
Estimated cumulative prosthetic survival for early and current designs. Number of patients still at risk of experiencing the primary endpoint and prosthetic survival with 95% CI per 5-year period are indicated in the life table

For early and current designs 10-year TAR survival was 0.69 (CI 0.64–0.73) and 0.84 (CI 0.79–0.88) respectively (Figure 2). Log rank test revealed a statistically significant difference in TAR survival between early and current designs in favor of current designs (p < 0.001) (Figure 2).

**Figure 3.**
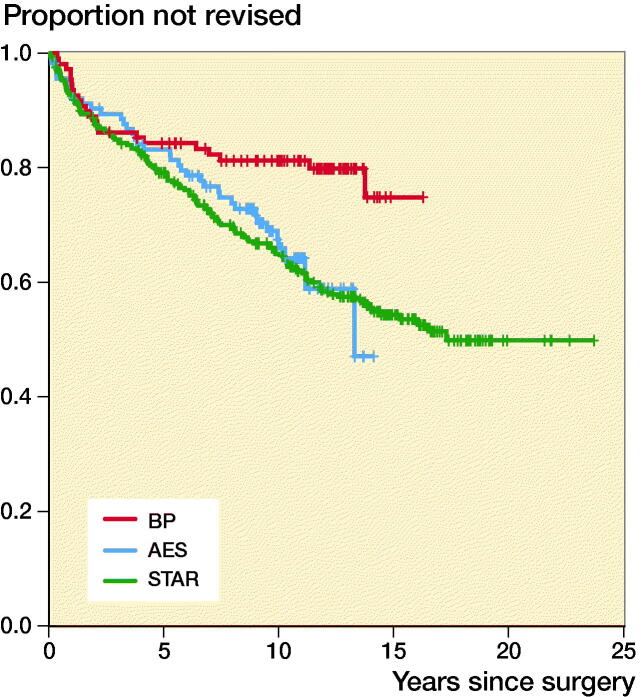
Estimated cumulative prosthetic survival for early designs. Number of patients still at risk of experiencing the primary endpoint and prosthetic survival with 95% CI per 5-year period are indicated in the life table.

**Figure 4.**
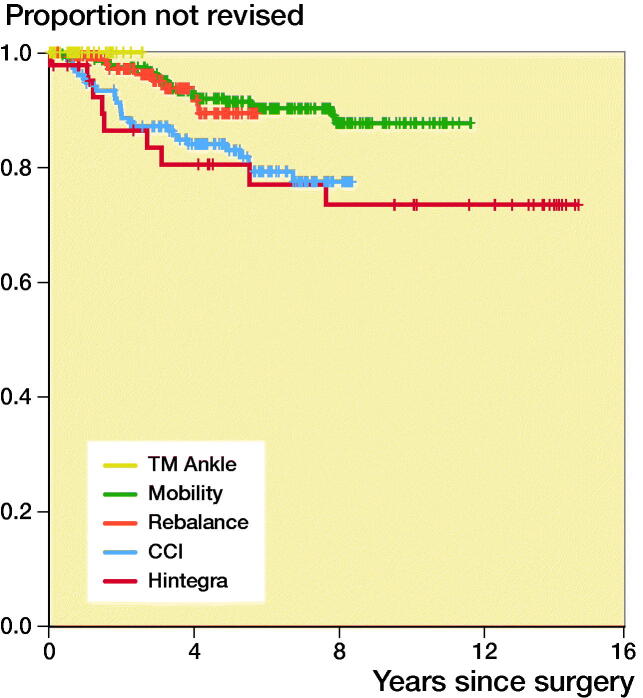
Estimated cumulative prosthetic survival for current designs. Number of patients still at risk of experiencing the primary endpoint and prosthetic survival with 95% CI per 5-year period are indicated in the life table.

Analyses by specific prosthetic design revealed a 5-year TAR survival rate of 0.89 (CI 0.82–0.97) for the currently most implanted design in Sweden, Rebalance. At the time of writing, no revision of TM Ankle design had been reported to the registry (Figures 3 and 4).

We found similar TAR survival rates (ranging from 0.57 to 0.69 at 15 years) for different diagnoses (Figure 5, see Supplementary data).

## Discussion

Using the Swedish Ankle Registry, we have demonstrated that survival of current TAR prostheses is higher than for earlier designs. In previous reports from our national registry the 5-year TAR survival rates were 0.78 for procedures made from 1993 to 2005 (Henricson et al. 2007) and 0.81 for 1993 to 2010 (Henricson et al. 2011b) respectively. In the current report we found the corresponding survival to be 0.85, indicating an improvement over time. This notion is further supported by 10-year TAR survival of 0.74 in the current study (Table 4, see Supplementary data) and 0.69 in the previous (Henricson et al. 2011b) (procedures undertaken from 1993 to 2010). In Sweden today, fewer units and surgeons perform TAR compared with earlier, resulting in larger volumes per surgeon. This and the presumed growing experience of these surgeons associated with time, may have contributed to the improving results.

**Table 3b.** Information on reasons for revision for current prosthetic designs

Reasons for revision	Hintegra n = 48	Mobility n = 269	CCI n = 151	Rebalance n = 178	TM ankle n = 37	Total (%)n = 683
Talus and/or tibia loosening	4	8	18	5		35 (49)
Technical error	2		1	1		4 (6)
Instability	1	4	1	1		7 (10)
Infection	1	1	1			3 (4)
Intractable pain		6	4	1		11 (15)
PE failure		1				2 (3)
Painful varus		1	2	1		4 (6)
Painful valgus	1	1				2 (3)
Fracture/Lux		1				1 (1)
Other		2	1			2 (3)
Total number of revisions (%)	9 (19)	25 (9)	28 (19)	9 (5)	0 (0)	71 (10)

a The inferior results of the STAR prosthesis is documented in previous reports from the Swedish Ankle Registry (Henricson and Carlsson 2015). The high frequency of “technical errors” may partly be explained by suboptimal instrumentation, limited experience of surgeons, and technical demands (Henricson et al. 2011b).

Reports from other national registries, published between 2007 and 2017, have presented 5-year survival rates between 0.78 and 0.89 and 10-year rates between 0.69 and 0.83, thus comparable to our findings, although the definition of revision is not always identical to our report (Table 4, see Supplementary data). In comparison with nationwide registries, results from single-center specialized units are often better (Labek et al. 2011), but have seldom been reproduced in countries where national registry data are available. Several such studies report 15-year survival rates between 0.64 and 0.76 (Table 4). This may be due to larger volumes of TARs per surgeon and also case mix, and supports the notion that TAR surgery takes a long time to master (Henricson et al. 2014, Barg et al. 2015).

Follow-up of 15 years or more has to our knowledge not previously been published by any other national registry. We found TAR survival rates at 15 years of 0.63 and at 20 years of 0.58. It is perhaps encouraging that long-term survival beyond 15 years is good with little change in survival between then and 20 years, although this inference is drawn from few patients. If we compare our 15-year TAR survival rates with 0.80 after knee arthroplasty and 0.88 after hip arthroplasty (Koskinen et al. 2008, Makela et al. 2014), the results are still, as previously noted, clearly inferior (Labek et al. 2011).

The general trend seems to be an increase in the use of TAR for treatment of ankle arthritis (Zaidi et al. 2013, Rybalko et al. 2018), although we did not find this to be true in Sweden. During 2016, 53 total ankle replacements were performed in Sweden (10 million inhabitants, 9 million inhabitants ≥ 15 years) indicating 0.6 replacements per 10^5^ inhabitants over the age of 15, a decrease compared with 1/10^5^ during 2011 when the incidence had plateaued (Henricson et al. 2011b). The low numbers in Sweden may partly be explained by logistical factors and staffing shortage in operating units and local procurement of prosthetic designs during recent years. A tendency of fewer patients with RA requiring TAR has also been noted during recent years, perhaps representing benefits of better non-surgical treatment.

For the early and current prosthetic design groups, 10-year TAR survival rate was 0.69 (CI 0.64–0.73) and 0.84 (CI 0.79–0.88) respectively (Figure 2). This may in part be referred to better prosthetic designs including instrumentation, but also to increasing surgeon experience, more careful patient selection, and improved healthcare.

Concerning separate prosthetic designs, we found that the currently most implanted models in Sweden (TM Ankle and Rebalance) show promising short-term results. Harris et al. (2014) studied 220 Rebalance prostheses, and similarly found encouraging early results. Some of the early models, such as AES, were withdrawn due to higher than expected complication rates (Di Iorio et al. 2017, Koivu et al. 2017b). Robust long-term follow-up results are important.

The most common reason for revision was loosening of the tibial and/or the talar component, accounting for about half of the revisions in both the early design group and current design group. Loosening has also been found to be the most common complication in several other studies from national registries as well as specialized/high-volume units (Barg et al. 2015). Polyethylene failure was the second most common complication in the early design group, whereas this complication was reported only once in the current design group. This may indicate improvement in implant design or manufacturing but may also partly be the result of longer follow-up of earlier designs. Further in-detail examination is necessary, preferably in a collaboration between several national registries.

In Sweden, available options of prosthetic designs are dictated by local procurement arrangements, which are based on local review of scientific reports and reports from the industry. This underlines the importance of future independent real-world studies from national registries.

The strength of national registry data is the real-world representation, i.e., data from several different surgeons and units, thus giving a picture of actual results in contrast to reports from single units or surgeons. Furthermore, this study has to our knowledge the hitherto largest cohort and follow-up time from a national ankle registry. Age, sex, and primary diagnosis seem overall to be similar to what has been reported in the literature.

The generalizability of our results may be limited. Data are derived from the specific setting of the Swedish Ankle Registry and results must be interpreted with care. However, results may still be applicable to similar populations (Table 2).

Another limitation of registry data is the uncertainty as to whether reporting is accurate and complete. We are confident that the reporting of primary TARs and revisions to the Swedish national ankle registry are accurate as the data are continuously compared with official data from the Swedish National Patient Registry by personal identity number and because the community of surgeons performing TARs is small. Furthermore, prosthetic failure was estimated from the date of the revision surgery, not from the date when failure was established. In addition, only revisions are reported, not failed implants that have not been revised. Given the observational study design, we cannot draw any causal inferences.

In conclusion, use of current prosthetic designs was associated with better TAR survival. This may in part reflect better prosthetic designs and instrumentation, but also increasing surgeon experience and better patient selection, as well as improved healthcare. However, TAR has a long way to go to approach survival rates for hip and knee arthroplasty.

## Supplementary data

Figure 5 and Table 4 are available as supplementary data in the online version of this article, http://dx.doi.org/10.1080/17453674.2019.1709312

## Supplementary Material

Supplemental Material
