# Supplementary material for: Better implant survival with modern ankle prosthetic designs: 1,226 total ankle prostheses followed for up to 20 years in the Swedish Ankle Registry
Source: Acta Orthop. 2020 Jan 13;91(2):191–6. doi: 10.1080/17453674.2019.1709312 (PMC7144194; doi:10.1080/17453674.2019.1709312)
Supplement: Supplemental Material [file IORT_A_1709312_SM0076.pdf]

Supplementary data

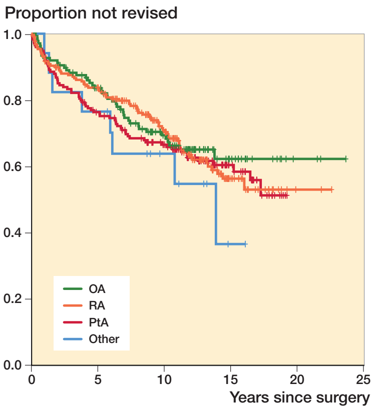

Figure 5. Estimated cumulative prosthetic survival per diagnosis. Number of patients still at risk of experiencing the primary endpoint and prosthetic survival with 95% CI per 5-year period are indicated in the life table.

Years since surgery:	5	10	15	20
Posttraumatic arthritis (PtA) (n = 441)				
Number at risk	243	106	31	
TAR survival	0.82	0.73	0.65	
CI	0.78–0.86	0.68–0.78	0.57–0.73	
Rheumatoid arthritis (RA) (n = 401)				
Number at risk	257	134	25	2
TAR survival	0.87	0.73	0.57	
CI	0.84–0.91	0.68–0.79	0.47–0.66	
Osteoarthritis (OA) (n = 291)				
Number at risk	163	74	18	3
TAR survival	0.87	0.75	0.69	
CI	0.83–0.91	0.69–0.82	0.59–0.78	
Other [a] (n = 93)				
Number at risk	44	11	1	
TAR survival	.76	0.65		
CI	0.66–0.86	0.48–0.81		

[a] Including hemophilia, hemochromatosis and psoriatic arthritis

Table 4. Prosthetic survival rates from national registries and high volume units

Study	Period of primary surgery	TAR (n)	Follow-up, years Mean	Max	Revisions (n)	TAR survival 5-year	10-year	15-year	20-year
Registry studies									
Fevang et al. 2007	1994–2005	257	3	12	27	0.89	0.76		
Henricson et al. 2007	1993–2005	531			101	0.78			
Henricson et al. 2011b	1993–2010	780	6	10	168	0.81	0.69		
Skytta et al. 2010	1997–2006	515	3	10	59	0.83			
Hosman et al. 2007	2000–2005	202	2	6	14	0.86			
http://www.nzoa.org.nz	2000–2017	728		11	50		0.83		
Undén et al. 2019 [a]	1993–2016	1,226	7	24	268	0.85	0.74	0.63	0.58
Non-registry studies									
Barg et al. 2013	2000–2010	722	6	10	61	0.94	0.84		
Bonnin et al. 2011	1997–2000	98	9	11	12		0.85		
Clough et al. 2019	1993–2000	200	16	24	32	0.90	0.83	0.76	
Frigg et al. 2017	1996–2006	50	15	16	12		0.78	0.70	0.55 [b]
Kerkhoff et al. 2016	1999–2008	134		15	20		0.78		
Koivu et al. 2017a	1997–2002	34		17	15	0.94	0.87	0.64	
Mann et al. 2011	1998–2000	84	9	11	9	0.96	0.90		
Palanca et al. 2018	1998–2000	84	16	18	15			0.73	
Wood and Deakin 2003	1993–2000	200	7	13	24	0.93	0.80		

[a] Current study
[b] At 19 years
